# Investigating the reliability of the evoked response in human iPSCs-derived neuronal networks coupled to micro-electrode arrays

**DOI:** 10.1063/5.0174227

**Published:** 2023-12-20

**Authors:** Giorgia Zanini, Giulia Parodi, Michela Chiappalone, Sergio Martinoia

**Affiliations:** 1 Department of Informatics, Bioengineering, Robotics, and Systems Engineering (DIBRIS), University of Genova, Genova, Italy; 2 IRCCS Ospedale Policlinico San Martino, Genova, Italy

## Abstract

*In vitro* models of neuronal networks have emerged as a potent instrument for gaining deeper insights into the intricate mechanisms governing the human brain. Notably, the integration of human-induced pluripotent stem cells (hiPSCs) with micro-electrode arrays offers a means to replicate and dissect both the structural and functional elements of the human brain within a controlled *in vitro* environment. Given that neuronal communication relies on the emission of electrical (and chemical) stimuli, the employment of electrical stimulation stands as a mean to comprehensively interrogate neuronal assemblies, to better understand their inherent electrophysiological dynamics. However, the establishment of standardized stimulation protocols for cultures derived from hiPSCs is still lacking, thereby hindering the precise delineation of efficacious parameters to elicit responses. To fill this gap, the primary objective of this study resides in delineating effective parameters for the electrical stimulation of hiPSCs-derived neuronal networks, encompassing the determination of voltage amplitude and stimulation frequency able to evoke reliable and stable responses. This study represents a stepping-stone in the exploration of efficacious stimulation parameters, thus broadening the electrophysiological activity profiling of neural networks sourced from human-induced pluripotent stem cells.

## I. INTRODUCTION

The brain stands as our body's ultimate enigma, with neuron interactions holding mysteries yet unraveled. While acquiring electrophysiological data *in vivo* remains challenging, *in vitro* neuronal networks present themselves as formidable assets, helping to shed light on the brain's intricate workings. Neurons, the functional units of the brain, communicate through the release of electrical and chemical stimuli, and it is widely recognized that endogenous electrical stimuli play fundamental roles in various biological phenomena, such as development, morphogenesis, cellular communication networks, and neuronal and glial cellular signaling (Adams and Levin, 2013; Baer and Colello, 2016; Bertucci *et al.*, 2019; Cao *et al.*, 2013; Forciniti *et al.*, 2014; and Nuccitelli, 2003). Understanding the complexities inherent in electrical stimulation assumes paramount significance while investigating neuronal signal processing across biological networks, thereby enhancing our grasp of synaptic integration mechanisms within individual neurons (Jimbo *et al.*, 2003). Furthermore, electrical stimulation delivered in the experimental setting can be used to study network connectivity, evaluate excitability, reduce variability, and replicate and modulate the neuronal activity (Pimashkin *et al.*, 2013; Jun *et al.*, 2007). Several works have shown that networks from rat primary neuronal cultures can adapt to external stimuli (Eytan *et al.*, 2003) and learn from them (Shahaf and Marom, 2001). Moreover, the firing of the bursting activity can be controlled by external stimuli and feedback (Wagenaar *et al.*, 2005), and low-frequency, uniform, sustained electrical stimulation can enhance the spontaneous firing rate and the bursting activity of neural cultures (Bologna *et al.*, 2010; Brewer *et al.*, 2009).

Among the several possibilities, the use of micro-electrode arrays (MEAs) to electrically stimulate *in vitro* networks has gained popularity, since it allows multi-site extracellular recording and noninvasive stimulation of the neuronal cultures in a controlled environment (Pelkonen *et al.*, 2022; Spira and Hai, 2013). To our current understanding, MEAs have predominantly found application in investigations involving *in vitro* neuronal networks derived from rodent sources, particularly in cases of localized stimulation (Massobrio *et al.*, 2015). Nevertheless, the results obtained from the studies of rodents-derived *in vitro* models cannot be directly translated to human-derived networks, which are becoming a standard nowadays (Lamas *et al.*, 2023). Indeed, human-induced pluripotent stem cells (hiPSCs) reproducing structural and functional aspects of the human brain are giving the possibility to obtain networks with the same phenotype of the donor, thus allowing the development of subject-specific networks. The systematic characterization and standardized electrical stimulation protocols for hiPSCs-derived neuronal networks are still lacking and, in turn, effective parameters to evoke reliable responses have not yet been identified. Consequently, a comprehensive characterization of the response elicited by electrical stimulation in a human-derived neuronal network is imperative.

To fill this gap, it is necessary to investigate the stimulation parameters able to (1) elicit evoked responses in the neuronal network and (2) guarantee stability and reliability of the responses. The choice of a stimulus that can elicit activity in neuronal cultures involves multiple variables. One crucial factor is the shape of the pulse, which can be controlled either by voltage or current. A study conducted by Wagenaar *et al.* (2004) proved that positive-then-negative biphasic voltage-controlled pulses are more effective compared to other pulse's shapes, indicating that voltage control may offer advantages in various stimulation scenarios. In most of the MEA-based literature, the pulse's shapes are characterized by phases of duration between 200 and 300 *μ*s (Li *et al.*, 2007) and by a peak-to-peak amplitude equal to 1.5 V (Vajda *et al.*, 2008; Zullo *et al.*, 2012). Another important aspect is the frequency at which the stimuli are emitted, since high or low frequency can lead to different results. The low-frequency stimulation (i.e., frequency range: 0–1 Hz) is usually exploited to understand whether and how the electrophysiological neuronal activity is affected by external stimulation, while the high frequency (i.e., frequency over 1 Hz) is generally used to manipulate the response of the neuronal networks by altering the connectivity between neurons (Le Feber *et al.*, 2010; Shahaf and Marom, 2001).

In this work, we investigated the stimulation parameters inducing independent and reliable evoked responses in human-derived neurons. We used biphasic voltage pulses at variable amplitudes (1.5, 2, 2.5, and 3 V) delivered at different frequencies (0.1 and 0.2 Hz). We applied low frequency stimulations to ensure we had independent responses, as demonstrated for primary rodent cultures; moreover, we used an amplitude not exceeding 3 V to avoid deterioration of the microelectrodes. As experimental model, we exploited glutamatergic (excitatory, E) and GABAergic neurons (inhibitory, I) to realize fully excitatory (E:I 100:0) and mixed (E:I 75:25) cortical networks derived from hiPSCs coupled to MEAs. We first designed the 100E configuration as it is the most prevalent and characterized one in the literature on networks derived from hiPSCs (Kirwan *et al.*, 2015). This configuration served as a straightforward model for conducting tests, without incorporating the complexity of mixed E/I cultures. Second, we developed and investigated the 75E25I networks as they represent the most similar model to the *in vivo* situation, in which the percentage of inhibitory neurons in the cortex and hippocampus ranges from 15% to 30% (Sahara *et al.*, 2012).

Our results indicate that human-derived networks typically present evoked responses much longer than primary cultures from rodents with a voltage stimulation that is more effective as the amplitude increases, reaching a good level of stability and reliability for amplitudes greater than those used in rodent networks (Chiappalone *et al.*, 2008; Wagenaar *et al.*, 2004). Our study represents a stepping-stone in the exploration of efficacious stimulation parameters, thus broadening the electrophysiological activity profiling of neural networks sourced from human-induced pluripotent stem cells.

## II. RESULTS

### A. Firing and bursting patterns

**FIG. 1.**
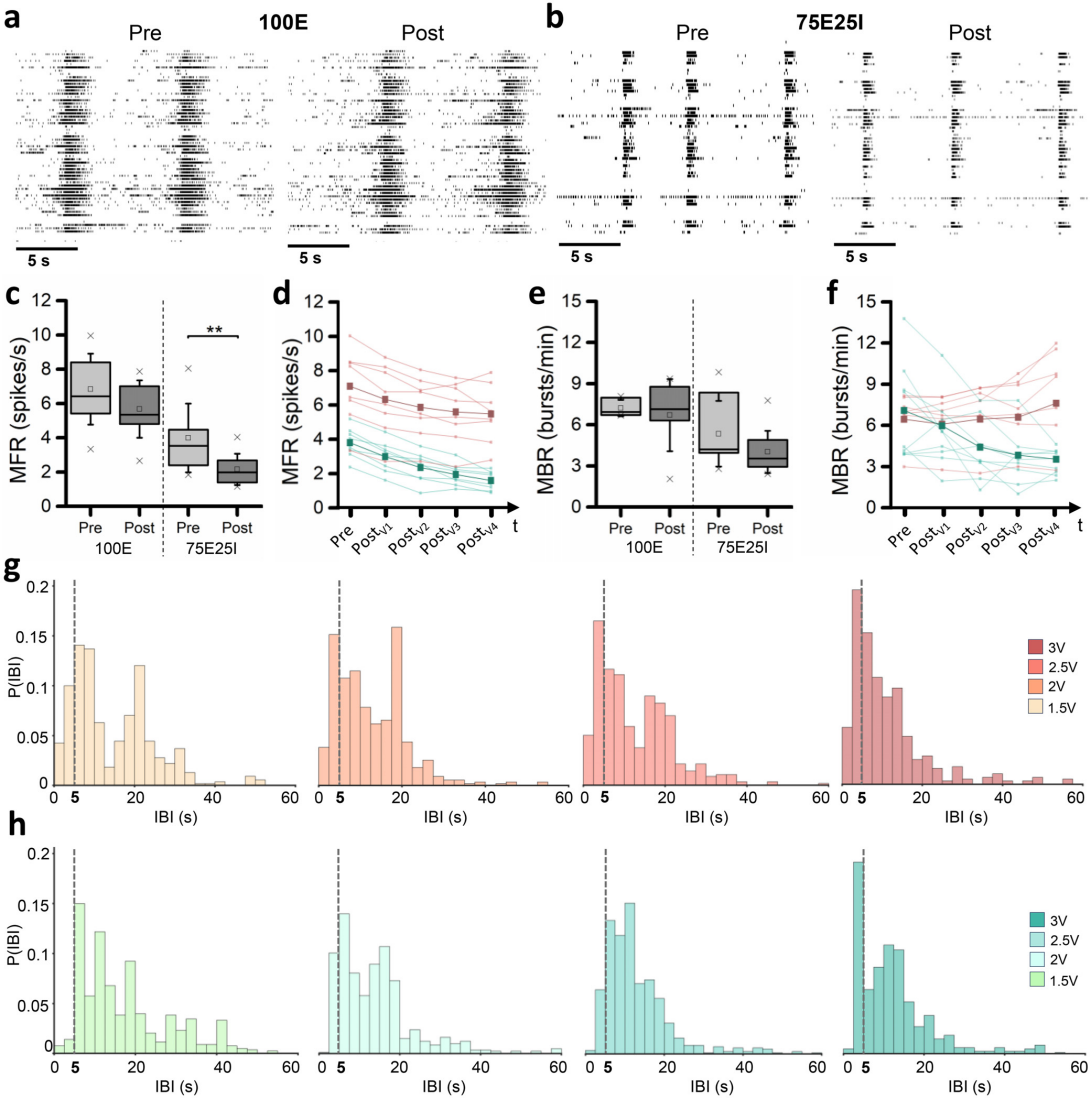
Firing and bursting pattern characterization. (a) Raster plots of a representative 100E neuronal network before (pre, left) and after (post, right) the stimulation. Each spike is represented with a small black bar, while a black band shows a network event. (b) Raster plots of a representative 75E25I neuronal network before (pre, left) and after (post, right) the stimulation. Each spike is represented with a small black bar, while a black band shows a network event. (c) Box plot of mean firing rate (MFR) pre- and post-stimulation of both 100E (left) and 75E25I (right) configurations. (d) MFR trend of each neuronal cultures of 100E (red) and 75E25I (green) configurations during the time in relation to each spontaneous-activity-phase E recorded. The thickest lines represent the average value of MFR of 100E (red) and 75E25I (green) configurations during the time. (e) Box plot of mean bursting rate (MBR) pre- and post-stimulation of both 100E (left) and 75E25I (right) configurations. (f) MBR trend of each neuronal cultures of 100E (red) and 75E25I (green) configurations during the time in relation to each spontaneous-activity-phase E recorded. The thickest lines represent the average value of MBR of 100E (red) and 75E25I (green) configurations during the time. (g) and(h) Histograms of the IBI distribution at each voltage amplitudes of 100E (g) and 75E25I (h) neuronal cultures, respectively. In the box plots, data are represented with the percentile 25–75 (box), the standard deviation (whiskers), the median (line), the mean (square), and the minimum and maximum (crosses) values (^**^ refers to p < 0.01).

To evaluate whether the electrical stimulation affected the vitality of the neuronal culture, we monitored the firing patterns before, during, and after the application of the stimulation protocol. From a qualitative point of view, we compared the raster plots of the same representative cultures before and after the stimulation [Figs. 1(a) and 1(b)], in which a network event can be noticed in the form of a dense black band. The 100E network is characterized by comparable raster plots before and after stimulation [Fig. 1(a)], showing network events with similar frequency, duration, and firing pattern. On the other hand, despite the 75E25I network showed a comparable network event frequency in the pre- and post-stimulation phase, a reduction of the firing can be noted by a less-dense black band [Fig. 1(b)]. By computing the MFR for the fully excitatory networks, we obtained comparable values before and after the stimulation phases [Fig. 1(c), Table S2] with no significant differences (Table S3). For the 75E25I configuration, we found a significant decrease in the MFR after the stimulation protocol [Fig. 1(c), Table S3], indicating that this configuration experienced more fatigue with respect to the fully excitatory networks in terms of sustained firing activity. To better understand this aspect, we reported the firing trend by showing the MFR separately for each culture in both configurations [Fig. 1(d)]. Independently from the stimulation order—since the four tested voltages were presented in a random order—the MFR values decreased during the time until the end of the stimulation protocol (50 min) for each network. Considering the bursting pattern, the cultures of both configurations showed comparable MBR values (Table S2) and a comparable trend with no statistical differences [Figs. 1(e) and 1(f), Table S4]. Since the frequency of the burst events did not show differences between the different voltages, we investigated whether the distribution in time of such events was affected by the voltages. We observed different IBI distributions and changings in their shapes by increasing the voltages of the stimulations. While at 1.5 V the IBI distribution of 100E showed somehow a bimodal distribution, the 75E25I presented a more spread distribution. By increasing the amplitude, the distribution became less broad with a clear peak around the stimulation interval (5 s), suggesting a redistribution of the burst events driven by the timing of the delivered stimuli.

### B. Network events and time-locking

**FIG. 2.**
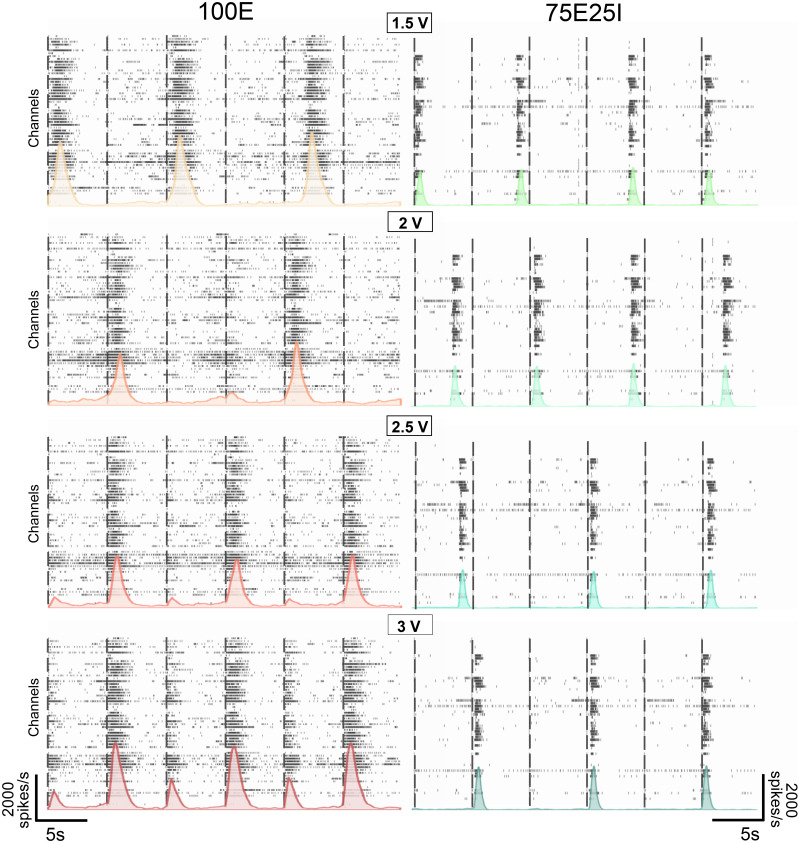
Qualitative evoked response evaluation. On the left: raster plots of 30 s of the electrophysiological activity of the same representative 100E neuronal culture, stimulated with a pulse amplitude equal to 1.5, 2, 2.5, and 3 V (from the top to the bottom). Each spike is represented with a small black bar, while a black band shows a network event. Overlapped to each raster plot, the cumulative instantaneous firing rate profile (bin = 10 ms) is reported. The dashed gray vertical lines indicate the time of stimuli emission. On the right: raster plots of 30 s of the electrophysiological activity of the same representative 75E25I neuronal culture stimulated with a pulse amplitude equal to 1.5, 2, 2.5, and 3 V (from the top to the bottom, respectively). Each spike is represented with a small black bar, while a black band shows a network event. Overlapped to each raster plot, the cumulative instantaneous firing rate profile (bin = 10 ms) is reported. The dashed gray vertical lines indicate the time of stimuli emission.

**FIG. 3.**
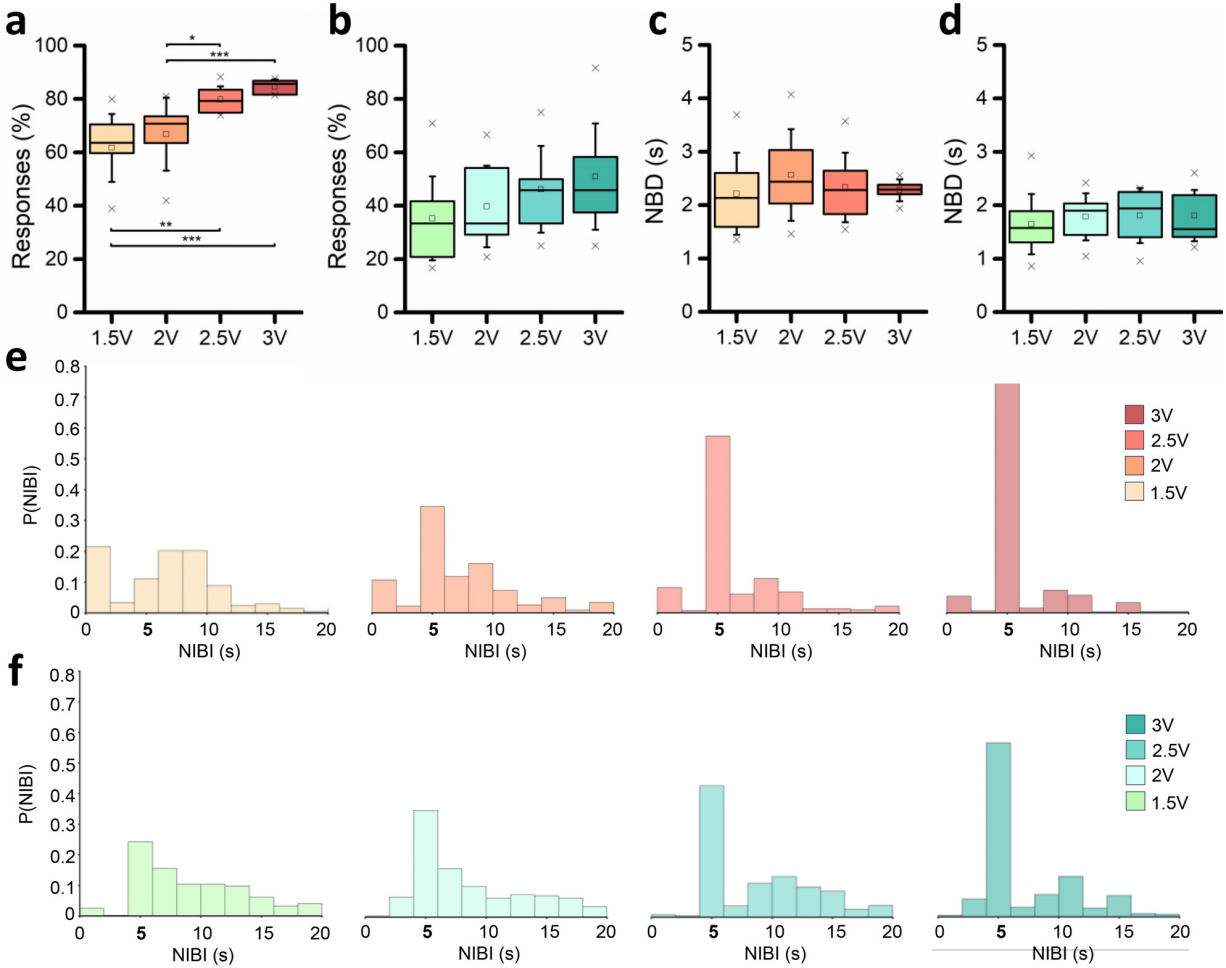
Network burst events characterization. (a)–(d) Box plots of number of positive responses to the stimulation of 100E (a) and 75E25I (b) configurations; network burst duration (NBD) of 100E (c) and 75E25I (d) configurations. (e) Histograms of the network-inter-burst-interval (NIBI) distribution at each voltage amplitudes of 100E neuronal cultures. (f) Histograms of the NIBI distribution at each voltage amplitudes of 75E25I neuronal cultures. In the box plots, data are represented with the percentile 25–75 (box), the standard deviation (whiskers), the median (line), the mean (square), and the minimum and maximum (crosses) values (^*^ refers to p < 0.05, ^**^ to p < 0.01, and ^***^ to p < 0.001).

Since we found differences in the burst event distributions, we investigated whether this difference propagated also at network events level. At first, we evaluated the evoked responses from a qualitative point of view, observing the raster plots and the cumulative histograms, highlighting the presence of the so-called “Network Burst” patterns. We reported the evoked activity of a representative culture stimulated at the different voltages from the same site for each configuration, i.e., 100E and 75E25I (Fig. 2). By increasing the voltage amplitude, a reorganization of the network bursting activity could be observed. The 100E network bursts were perfectly aligned with the stimuli when the neuronal network was stimulated with pulses of amplitude equal to 2, 2.5, and 3 V, while at a lower amplitude the bursts occurred almost independently of the timing of the stimuli, suggesting a non-totally effective stimulation. Furthermore, a major recruitment of the network could be observed as the voltage increased, by looking at both the raster plot and the histograms. Indeed, while three network bursts could be noticed in the reported examples of the evoked activity at 1.5, 2, and 2.5 V, three new events appeared at 2.5 V, and six network bursts were observed when stimulating at 3 V. For the 75E25I configuration, an almost reliable time-lock with the stimuli emission could be noted only for the 3 V stimulation, while at lower amplitudes, the networks events still partially occurred in an independent manner. Furthermore, no changes were observed in terms of number of generated network events, differently from the 100E configuration. To quantify all the above, the number of responses of the 100E cultures revealed statistical differences among almost all voltages [Fig. 3(a), Tables S5 and S7], showing values that approach the 80% and 100% for the 2.5 and 3 V stimulation, respectively, demonstrating a high recruitment rate of the network and the effectiveness of both the 2.5 V and (even more) the 3 V stimulation. Observing the number of responses related to the 75E25I configuration [Fig. 3(b), Table S5], the same increasing trend can be denoted, although the average number of responses approaches the 50% and did not show statistical differences as for the 100E configuration (Table S7). On the other hand, the network burst duration did not show statistical differences between the stimulation phases for both configurations [Figs. 3(c) and 3(d), Tables S5 and S6], supporting what we found for the bursting pattern (cf. Fig. 1). The redistribution of the network events was delineated by a different network-inter-burst-interval (NIBI) distribution [Fig. 3(d)]. Indeed, both 100E and 75E25I configurations showed a clear peak around the stimulation interval (5 s) that increased when the voltage amplitude increased, indicating the redistribution of the network burst events driven by the timing of the delivered stimuli and reaching the maximum peak amplitude of the distribution with the 3 V stimulation.

### C. PSTH and evoked response

**FIG. 4.**
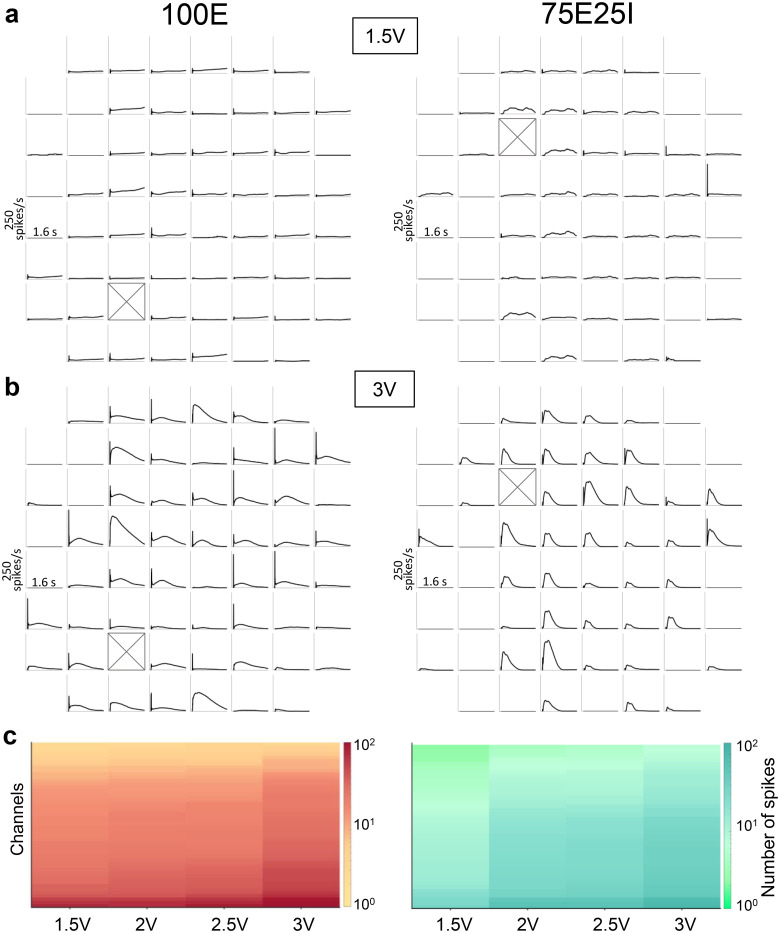
Evoked response of representative networks. (a) Response of 100E (left) and 75E25I (right) representative networks to 1.5 V stimulation. Each box represents one channel. The site of the stimulation emission is represented as a black box crossed. (b) Response of 100E (left) and 75E25I (right) representative networks to 3 V stimulation. Each box represents one channel. The site of the stimulation emission is represented as a black box crossed. (c) Color maps of the 100E (left) and 75E25I (right) neuronal networks representing the number of spikes detected in 2.4 ms after the stimuli emission for each tested amplitude.

**FIG. 5.**
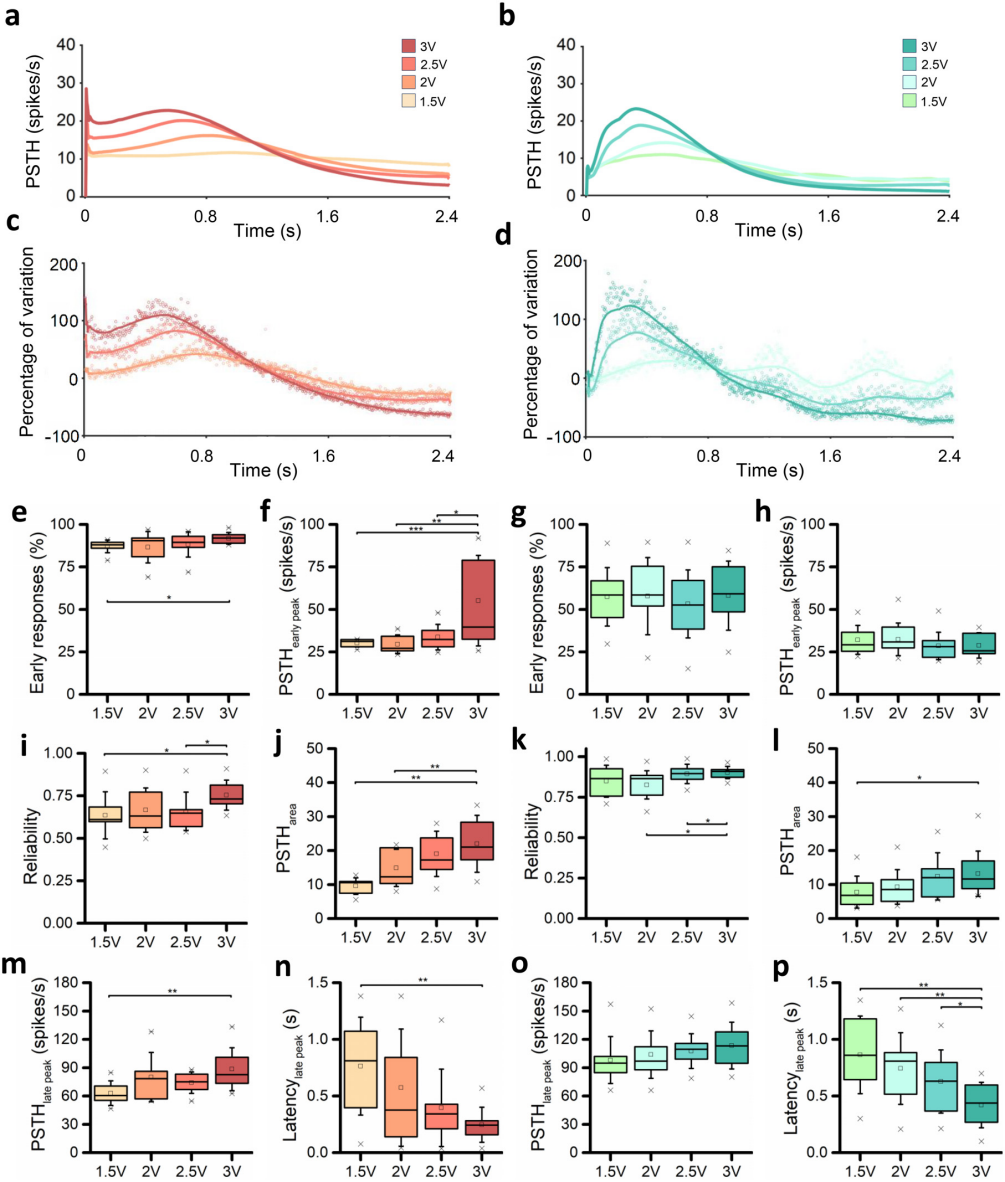
Evoked response characterization. (a) Average PSTH of the 100E neuronal networks stimulated with all the voltages. (b) Average PSTH of the 75E25I neuronal networks stimulated with all the voltages. (c) Percentage of variation between 2, 2.5, and 3 V with respect to the 1.5 V evoked response of the 100E configuration; (d) percentage of variation between 2, 2.5, and 3 V with respect to the 1.5 V evoked response of the 75E25I configuration. Box plots of (e) percentage of electrodes which showed an early response for the 100E configuration; (f) amplitude of the PSTH peak of the early response for the 100E configuration; (g) percentage of electrodes which showed an early response for the 75E25I configuration; (h) amplitude of the PSTH peak of the early response for the 75E25I configuration; (i) 100E reliability; (j) PSTH area of the 100E configuration; (k) 75E25I reliability; (l) PSTH area of the 75E25I configuration; (m) amplitude of the PSTH peak of the late response for the 100E configuration; (n) latency of the PSTH peak of the late response for the 100E configuration; (o) amplitude of the PSTH peak of the late response for the 75E25I configuration; and (p) latency of the PSTH peak of the late response for the 75E25I configuration. In the box plots, data are represented with the percentile 25–75 (box), the standard deviation (whiskers), the median (line), the mean (square), and the minimum and maximum (crosses) values (^*^ refers to p < 0.05, ^**^ to p < 0.01, and ^***^ to p < 0.001).

To evaluate the responses to the stimuli, we computed the PSTH (cf. Sec. IV E 2) for each channel of two representative cultures [Figs. 4(a) and 4(b), Fig. S2]. By increasing the voltage amplitude, the number of electrodes recruited in the evoked response increased, with a parallel increase in both PSTH peak and area, qualitatively observable. To prove the last assertion, we computed the number of spikes detected during the 2.4 s after the emission of the stimuli for the representative neuronal network. We reported the results in a color map reporting the number of evoked spikes per channel, to appreciate the differences between the four different stimulation amplitudes [Fig. 4(c)]. To evaluate the robustness of the data, we also evaluated the coefficient of variation (CV, Fig. S1) of the most representative feature of evoked response, i.e., the PSTH area. Since the stimulation phase has four different sites of stimulus' emission, we investigated the robustness of each culture's activity by computing the CV of the PSTH area among the phases. The latter showed lower values and no statistical differences, allowing us to average the features over all the phases, obtaining one representative value for each culture at each voltage amplitude. We then computed the average PSTH shapes [Figs. 5(a) and 5(b)] and the 90% confidence interval (CI_90%_) over all the cultures for each voltage (Fig. S3). The networks were characterized by repeatable neuronal activity, since the CI_90%_ of the average PSTH was restricted for both the configurations (Fig. S3). Qualitatively considering the PSTH shapes overlapped [Figs. 5(a) and 5(b)], a proportional increase in the curves' amplitude can be observed, both in terms of early (until 100 ms) and late (from 100 ms forward) responses. The decaying phase of the response for each configuration was comparable in time between the different voltages. To deepen this aspect, we considered the variation of percentage of the evoked response (2, 2.5, and 3 V) with respect to the 1.5 V stimulation. For both configurations, a precise time-point can be identified as the end of the stimulation effect. In particular, we empirically identified as end time of the stimulation effect 1.1 and 0.8 s for the 100E [Fig. 5(c)] and the 75E25I [Fig. 5(d)] configuration, respectively, and we computed the parameters related to the stimulation by considering the PSTH until the above-mentioned temporal points. The main difference between the two configurations was related to the presence of a first peak, i.e., an early response, in the 100E PSTH [Fig. 5(a)], that seems to almost disappear in the 75E25I configuration [Fig. 5(b)]. To quantify, we computed the percentage of early responses with respect to the active electrodes. While the 100E, for each voltage amplitude, showed values over the 75% [Fig. 5(e), Tables S8 and S9], approaching the 100% for the 3 V stimulation, the 75E25I displayed remarkably lower amounts of early responses, with average values close to the 50% for each voltage amplitude [Fig. 5(g), Tables S8 and S9]. This supports the 100E PSTH_early peak_ substantial differences found at 3 V with respect to 1.5, 2, and 2.5 V [Figs. 5(f), Table S10], proving the higher recruitment related to higher voltages while, due to the low amounts of early responses, the 75E25I PSTH_early peak_ did not display statical differences, showing a constant trend among the different voltages [Fig. 5(h), Table S10]. By focusing on the effect of the stimulation amplitude, an increase in the reliability can be observed for both 100E and 75E25I configurations [Figs. 5(i) and 5(k), Table S8], demonstrating with statistical differences the effectiveness of the 3 V stimulation (Table S11). This result can be further confirmed by the PSTH_area_, which showed significative differences for both configurations [Figs. 5(j) and 5(l), Table S12], and by the increasing amplitude of the PSTH_late peak_ [Figs. 5(m) and 5(o), Tables S8 and S13], followed by a decreased latency of the late peak [Figs. 5(n) and 5(p), Tables S8 and S14], proving a higher recruitment of the network.

### D. Different low frequencies did not change the effects of the stimulation

**FIG. 6.**
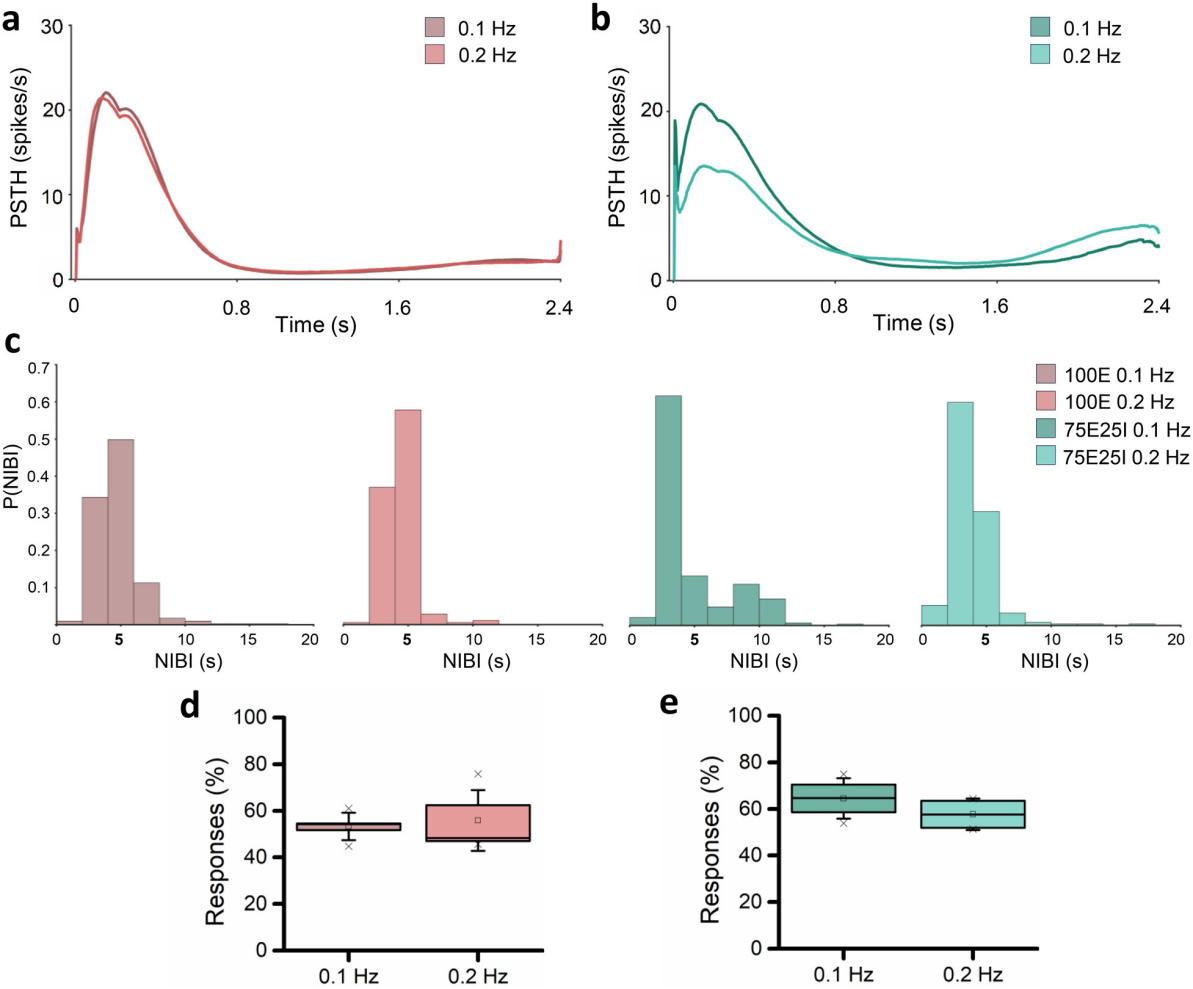
Characterization of the evoked response from 0.1 and 0.2 Hz stimulation. (a) PSTH of the 100E configuration stimulated with 0.1 and 0.2 Hz; (b) PSTH of the 75E25I configuration stimulated with 0.1 and 0.2 Hz; (c) histograms of the NIBI distribution for 100E (left) and 75E25I (right) neuronal cultures when stimulated at 0.1 and 0.2 Hz. Number of positive responses to the stimulation of 100E (d) and 75E25I (e) configurations. In the box plots, data are represented with the percentile 25–75 (box), the standard deviation (whiskers), the median (line), the mean (square), and the minimum and maximum (crosses) values.

To investigate whether different low frequencies affected the evoked response, we tested on the same neuronal cultures (i.e., five 100E and four 75E25I) the stimulation at two different low frequencies, i.e., 0.1 and 0.2 Hz. For the 100E configuration, the PSTHs obtained from both frequencies were extremely comparable, both in shape, amplitude, and duration, displaying no differences [Fig. 6(a)]. For the 75E25I, despite comparable shapes and durations, a difference in the amplitude of the PSTH could be noted [Fig. 6(b)]. Specifically, the PSTH amplitude increased with 0.1 Hz stimulation, likely due to delayed stimuli emission. This aided the network's recovery, leading to a stronger response to subsequent stimuli. In contrast, at 0.2 Hz, frequent electrical stimulation stressed the network, causing reduced response and recruitment. The distribution of the network events was delineated by similar NIBI distribution [Fig. 6(c)], with a clear peak around the stimulation interval (5 s), suggesting a distribution of the network burst events driven by the timing of the delivered stimuli for both 0.1 and 0.2 Hz stimulation. Finally, by observing the number of positive responses, both 100E and 75E25I configurations showed a highly comparable (Table S16) number of evoked responses for both 0.1 Hz and 0.2 Hz stimulation (Table S15), proving the effectiveness of both low frequencies.

## III. DISCUSSION

Our study aims to fill a gap in the characterization of the electrical stimulation of human-derived neurons coupled to MEAs. Since neuromodulation, in general, and electrical stimulation, in particular, are gaining an increasing role in the treatment of neurological diseases, a systematic investigation of the effect of electrical stimulation in human-derived neuronal cultures is mandatory. Indeed, recent studies showed how electrical stimulation could be employed to treat the pathological patterns of *in vivo* networks, such as in the case of Parkinson's disease, essential tremor, dystonia, and obsessive-compulsive disorder (Marín *et al.*, 2022). Nevertheless, before the *in vivo* application, testing on *in vitro* networks would help to improve the personalization strategy. To this end, it is important to emphasize the great benefit brought by the use of networks derived from human-induced pluripotent stem cells, which, being directly obtained from patients, allow to recreate *in vitro* networks with their same phenotype. For all these reasons, our study focused on the identification of the effective stimulation parameters for inducing activation in neuronal networks derived from hiPSCs. We examined the elicited electrophysiological responses associated with varying voltage levels and frequencies of stimulation, utilizing both fully excitatory and mixed (with a composition of 75% glutamatergic and 25% GABAergic) neuronal cultures. We deliberately chose neither low- nor high-neuronal density in order to stay at an intermediate level of cells. Our choice remained consistent across the tested configurations (both 100E and 75E25I), providing us with a uniform and comparable dataset in terms of cellular density. We performed the experiment at days *in vitro* (DIV) 56, a time point where the shift of GABAergic neurons had already been completed, thereby ensuring the presence of a fully developed inhibitory component (Mossink *et al.*, 2021).

**FIG. 7.**
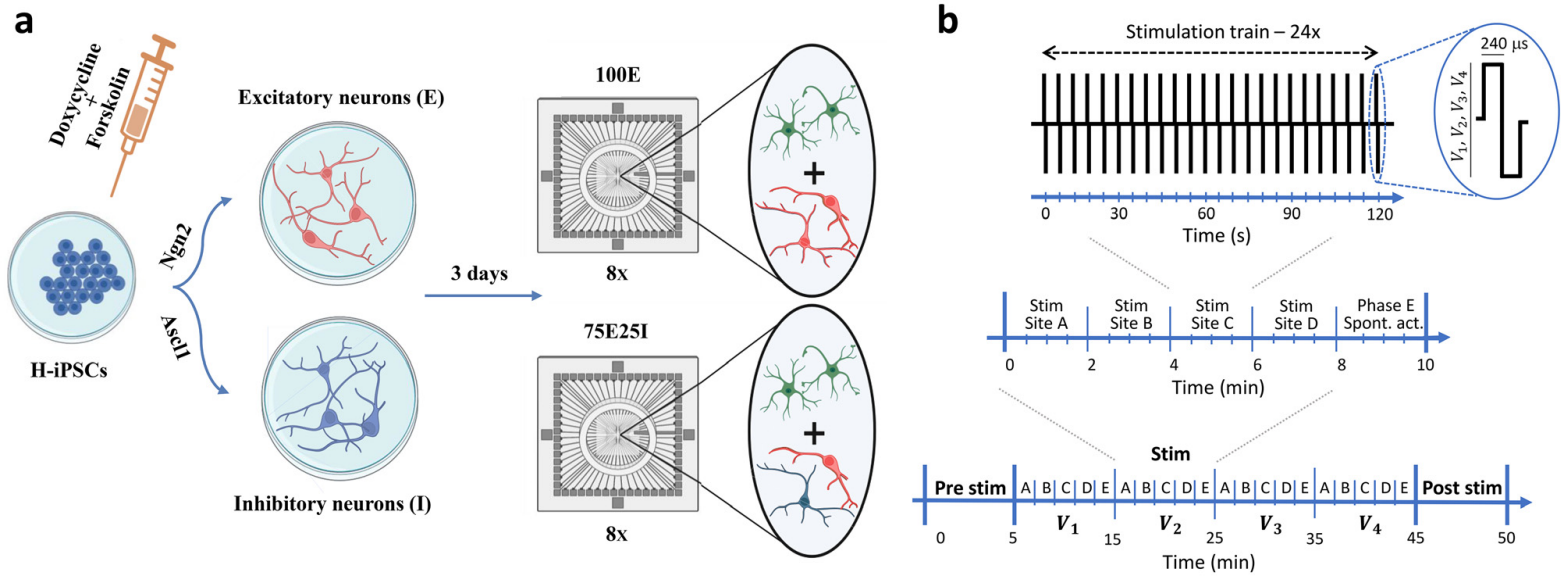
Experimental protocol. (a) Schematic representation of the neuronal cultures protocol. From left to right: hiPSCs were induced to differentiate into excitatory (in red) and inhibitory (in blue) neurons by adding doxycycline and forskolin compounds to the culture medium. After 3 days, the neurons (N) were moved to MEAs and mixed with rat astrocytes (A, in green) to obtain cultures composed of 70% of neurons and 30% of astrocytes. For the 100E configuration, we plated exclusively glutamatergic neurons, for the 75E25I configuration we mixed glutamatergic (75%) and GABAergic (25%) neurons. (b) Schematic representation of the applied stimulation protocol. From the top to the bottom: a 2-min stimulation train composed of 24 biphasic stimuli (240 *μ*s per pulse phase) was emitted at one of the different voltages' amplitudes (i.e., 1.5, 2, 2.5, and 3 V). Each train was delivered from four different sites (e.g., site A, site B, site C, and site D), followed by a 2-min recording of spontaneous activity (phase E). This phase was repeated for each stimulation amplitude.

From the wide variety of possible stimulation pulse shapes that can be applied to MEA electrodes, we explored biphasic rectangular current pulses of polarity positive–negative, accordingly on what was found in rodents' cultures (Wagenaar *et al.*, 2004). We tested different pulses' amplitudes, i.e., 1.5, 2, 2.5, and 3 V (Fig. 7) and two different frequencies (0.1 and 0.2 Hz) to understand which stimulation parameters mostly affected the evoked response. Regarding the frequencies, the ones we adopted are in line with the conventional “test stimulus” approach, which draws inspiration from pioneering studies in the MEA field (Jimbo and Kawana, 1992). This approach was primarily designed to simply probe the network and assess its elicited response. Furthermore, the chosen stimulation frequencies, set at 0.2 and 0.1 Hz, are notably low and are unlikely to induce any plastic change within the network, as supported by prior investigations involving rodent models (Chiappalone *et al.*, 2008; Shahaf and Marom, 2001). Indeed, we found that the efficacy of the stimulation in both plating conditions (i.e., 100E and 75E25I) was primarily related to the amplitude of the pulses, with no significant impact observed from varying low frequencies. Notably, a 3 V stimulation consistently yielded the best results in terms of recruitment and the number of evoked responses.

As a general outcome, we found that the human-based experimental model greatly differs from the rodent-based one in terms of evoked activity. This is well in line with the recent outcomes by previous study (Estévez-Priego *et al.*, 2023), which underlined the differences between human-derived and rodents' cultures, exhibiting the richer hiPSCs activity patterns with respect to rat primary cultures. In our work, we observed that human-derived networks exhibited substantially prolonged responses, extending for approximately 2 s. In contrast, networks derived from rodents typically terminated their responses within a considerably shorter time frame of around 500–600 m (Chiappalone *et al.*, 2008). Furthermore, human-derived networks did not show reliable responses to low voltages, which are, instead, standard for rodents (i.e., 1.5 V) (Kapucu, 2012; Wagenaar *et al.*, 2004). We found all the above for both the tested configurations, i.e., 100E and 75E25I, but with important differences. Indeed, it is worth to underline that the configuration closest to *in vivo*, that is the mixed one, showed a slightly reduced firing after extensive stimulation and a behavior qualitatively similar to the primary rodents' cultures even if with the need for an increased voltage amplitude and a prolonged evoked response. Being a long stimulation protocol, the cells suffered the stress of stimulation, decreasing the firing during time, both for the 100E and for the 75E25I, but the latter with significant difference. Despite this, both configurations showed a redistribution of bursting patterns (Figs. 3 and 3), locking the events timing in a 5 s window (i.e., time delivery of stimuli), both at the single channel and network level. For 100E, the effectiveness of stimulation can already be observed at 2 V, where network events began to synchronize with the stimuli, while at 1.5 V the network events seemed to be independent and autonomous with respect to the stimulation. By increasing the amplitude of the voltage, we noticed an increase in the network recruitment as both the frequency and the amplitude of the network events increased (Figs. 4 and 5). For the 75E25I, we can assert the same considerations, but with a lower recruitment, as the 75E25I showed only 50% of evoked responses (one in two stimuli). Indeed, being GABAergic neurons present in the cultures, an inhibitory action is carried out in the network, thus slowing down the resumption of culture before the emission of a new network event.

As for the tested frequencies, we showed that 0.1 or 0.2 Hz did not cause any difference for the 100E (Fig. 6). On the other hand, since the 75E25I responded one stimulus in two with the 0.2 Hz stimulation, as we expected, there were no changes in the number of responses with the 0.1 Hz stimulation. However, there was an increase in the area and peak of the PSTH with 0.1 Hz: the network had more time to recover, leading to a greater recruitment of the network, which results in a wider response (Fig. 6).

In the future, it would be interesting to assess the efficacy of electrical stimulation in chemically modulated networks. This would help us delve deeper into understanding the functional significance of the E/I (un)balance (Parodi *et al.*, 2023). To specifically demonstrate the operational role of inhibition, it may be feasible to hinder the network by employing saturating doses of bicuculline, as demonstrated in previous studies (Soriano *et al.*, 2008; Tibau *et al.*, 2013). Such an approach would enable us to compare the response of a fully excitatory network with that of a physiological network that has experienced a reduction in inhibition. In conclusion, our study demonstrates that neuronal networks derived from hiPSCs can be reliably electrically stimulated, given the provision of appropriate parameters. The effectiveness of biphasic pulses exhibiting positive–negative polarity has been confirmed in these cultures, extending to both rodent-derived cultures and human-derived ones. Our findings establish that the stimulation outcome depends more on pulse amplitude rather than frequency. To ensure consistent results, a minimum of 2.5 V is required for stimulation, with the optimal amplitude being 3 V. However, it is worth noting that these amplitudes might need adjustment based on electrode area and material. As a result, we have established protocols and parameters that can be conveniently applied to human-derived neuronal cultures, opening up new horizons in the realm of personalized medicine.

## IV. METHODS

### A. HiPSCs cultures maintenance

We used two previously characterized hiPSCs lines genetically modified to obtain homogeneous populations of excitatory and inhibitory neurons [Fig. 7(a)], thanks to the forced expression of the transcription factors Neurogenin-2 (Ngn2) and Achaete-scute homolog 1 (Ascl1) (Mossink *et al.*, 2021). We received the hiPSCs lines in frozen vials from Dr. Frega (University of Twente). Both lines were generated from fibroblasts. Glutamatergic neurons were derived from control line 1 (C1, healthy 30-year-old female, Ngn2), which was reprogrammed via episomal reprogramming (Coriell Institute for Medical Research, GM25256). GABAergic neurons were derived from control line 2 (C2, healthy 51-year-old male, Ascl1), which was reprogrammed via a non-integrating Sendai virus (KULSTEM iPSC core facility Leuven, Belgium, KSF-16-025). Cultures were thawed and maintained in E8 Flex medium (Thermo Fisher Scientific) supported with the following compounds: E8 supplements (2%, Thermo Fisher Scientific), penicillin/streptomycin (1%, Sigma-Aldrich), G418 (50 *μ*g/ml, Sigma-Aldrich), and puromycin (0.5 *μ*g/ml, Sigma-Aldrich). Glutamatergic cortical Layer 2/3 neurons were derived from C1 by overexpressing mouse neuronal determinant Ngn2 upon doxycycline (4 *μ*g/ml, Sigma-Aldrich) treatment (Frega *et al.*, 2017). GABAergic neurons were derived from C2 by overexpressing mouse neuronal determinant Ascl1 (Addgene 97329) upon doxycycline treatment and supplementation with forskolin (10 *μ*M, Sigma Aldrich) (Mossink *et al.*, 2021). The hiPSCs can be considered differentiated into neurons after 3 weeks of doxycycline and forskolin treatment (Frega *et al.*, 2017; Shi *et al.*, 2016). The cultures were preserved into the incubator at stable condition (37 °C, 5.5% CO_2_, 95% humidity atmosphere). The 50% of the medium was refreshed every 2 days.

### B. Neuronal networks plating and maintenance

The neuronal networks were treated with the adapted pipeline presented in Wang *et al.* (2023). Briefly, PDMS rings were utilized to both confine and protect the cells without creating patterned networks and affecting the neurons' viability (Liu *et al.*, 2019; Ming *et al.*, 2021). Each ring had a diameter of 6 mm and height of about 1 mm. The PDMS rings were obtained using a mixture of PDMS base (Sylgard 184) and curing agent at a 10:1 (w/w) ratio, which was polymerized in oven (80 °C for 15 min). The day before the neuronal cultures plating, the rings were positioned and let adhere to the Micro-Electrode Arrays (MEAs, Multi Channel Systems – MCS, Reutlingen, Germany) surface. The devices were sterilized in an oven (120 °C for 3 h) and pre-coated over night with poly-L-ornithine (50 *μ*g/ml, Sigma-Aldrich) and human laminin (20 *μ*g/ml, BioLamina). At days *in vitro* (DIV) 0, neuronal cultures were detached with Accutase (Sigma-Aldrich) and co-plated on the pretreated devices with rat astrocytes to favor the growth and the maturation of the neurons (Tang *et al.*, 2013). For what concerns the purely excitatory networks, we plated exclusively glutamatergic neurons, and we will refer to this configuration as 100E. On the other hand, to obtain the E:I 75:25 configuration, we mixed glutamatergic (75%) and GABAergic (25%). We will refer to this configuration as 75E25I. The neuronal cultures density was set to 1200 cells/mm^2^. During the first week, the neuronal cultures were maintained in neurobasal medium (Thermo Fisher Scientific) enriched with the following supplements: B27 supplements (2%, Thermo Fisher Scientific), penicillin/streptomycin (1%, Sigma-Aldrich), stable L-glutamine (1% GlutaMax 100×, Thermo Fisher Scientific), human brain-derived neurotrophic factor (BDNF, 10 ng/ml, Sigma-Aldrich), human neurotrophin-3 (NT-3, 10 ng/ml, Sigma-Aldrich), doxycycline (4 *μ*g/ml, Sigma-Aldrich), and forskolin (4 *μ*g/ml, Sigma-Aldrich). At DIV 7, we added fetal bovine serum (FBS, 2%, Thermo Fisher Scientific) to the aforementioned supplemented medium to support astrocytes. At DIV 14, we removed doxycycline and forskolin from the medium. The neuronal cultures on MEAs were stably maintained in the incubator up to 56 DIV. The 50% of the medium was changed every 2 days.

### C. Stimulation protocol

To perform the electrical stimulation, we tested biphasic (positive-then-negative) pulses of various amplitudes (i.e., 1.5, 2, 2.5, and 3 V peak-to-peak) with a duration equal to 240 *μ*s per phase and a frequency of 0.2 or 0.1 Hz. First, we recorded the spontaneous electrophysiological activity for 5 min. During this phase, we chose four electrodes that showed sustained spiking and bursting activity for each culture, indicating that they were connected to the rest of the network and participated in the collective activity (Chiappalone *et al.*, 2008); these electrodes were then used as stimulation sites. Subsequently, we stimulated the neuronal networks with one of the tested amplitudes from the previously chosen sites of stimulation and we recorded the evoked activity for 2 min [cf. Fig. 7(b)]. We repeated this stimulation from the four electrodes. We indicate this phase of the protocol as “stimulation phase.” Each stimulation phase was interposed by 2-min recordings of the spontaneous activity (phase E) and characterized by a different pulses' amplitude. The pulse amplitudes were delivered in a random sequence to avoid the association of the evoked response to the order of the presented stimuli. The protocol's sequences are reported in Fig. 7(b).

### D. Dataset and recordings

In order to have stable culture conditions, the experiments were performed with the MEA2100 recording system (MCS) after the GABA switch (Mossink *et al.*, 2021), between DIV 56 and DIV 70. After a 10-min period of accommodation outside the incubator, the neuronal activity was recorded at a sampling frequency of 10 kHz in stable environmental condition (37 °C, 5% CO_2_), according to the stimulation protocol described in Sec. IV C. The electrophysiological activity was recorded from all the 59 electrodes (i.e., the remaining one is the reference electrode) of the MEAs. The dataset was composed of eight 100E (purely excitatory) and eight 75E25I (mixed physiological) neuronal cultures.

### E. Data analysis

In Subsections IV E 1–IV E 3, we present in detail the analyses carried out on the data and the parameters used to characterize the networks. These parameters are summarized in Table S1.

#### 1. Spiking and bursting activity

Data analyses were performed using SpyCode (Bologna *et al.*, 2010) and in-house code developed in MATLAB (The MathWorks, Natick, MA, USA), allowing the extraction of features that describe the spontaneous and the evoked network electrophysiological activity. We pre-processed the data adapting the pipeline presented in a previous study (Brofiga *et al.*, 2022). Briefly, we performed the spike detection using the Precision Timing Spike Detection (PTSD) algorithm (Maccione *et al.*, 2009). Then we set the following parameters required from the PTSD: peak life period (= 2 ms), related to the duration of the spike, and refractory period (= 2 ms), i.e., the minimum time elapsed between two consecutive spikes. The noise threshold for individual electrode was set at 10 times the standard deviation of the baseline noise. We computed the mean firing rate (MFR) as the number of spikes detected in the unit of time. Channels with MFR < 0.1 spikes/s (sp/s) were considered inactive (Mossink *et al.*, 2021). Noisy electrodes were discarded at the before starting the recording session. For each considered culture, the MFR was computed by averaging the firing rate of each active channels. Burst detection was performed with the logarithmic binning of the inter-spike interval (ISI) (Pasquale *et al.*, 2010). We considered a burst event if the following conditions were met: activity composed of at least ten consecutive spikes with an inter-burst interval lower than 50 ms. We computed the mean bursting rate (MBR) as the number of bursts per minute. An active channel with bursting rate was greater than 0.4 bursts/min was considered as bursting channel (Mossink *et al.*, 2021). For each culture, the MBR was calculated by averaging the bursting rates of each bursting channel. An event was defined as network burst if 20% of the bursting channels simultaneously presented activity. From the network burst detection, the network burst duration (NBD) was computed.

#### 2. Evoked activity

The post stimulus time histograms (PSTHs) were calculated by considering a 2.4 s time window after the stimulus emission. In particular, we divided each time window into 4 ms bins and counted the number of spikes occurring in each bin. We discarded the PSTHs characterized by an area (i.e., total number of evokes spikes) lower than 4 since these stimuli were considered unable to evoke a response. We computed the percentage of effective stimuli, able to evoke a response over a total of 24 stimuli for each stimulation site. We obtained the PSTH of each culture by averaging the single-channel PSTHs. We can divide the evoked response into three main components: an early response (the fastest component, which typically occurs in the first 100 ms and which includes the direct activation of neurons by the stimulation), a refractory period (period with a reduced firing probability), and a late response (the slowest component starting after the first 100 ms) (Jimbo *et al.*, 1999; Marom and Shahaf, 2002; and Shahaf and Marom, 2001). To quantify the above typical responses, we first evaluated the percentage of electrodes that exhibited an early response by assessing whether the PSTH was characterized by a peak during the first 100 ms. The maximum value of the curve of PSTH in the early phase was identified as “PSTH_early peak_.” Similarly, we computed the timing at which the peak of the late response was detected, identified as the “Latency_late peak_” and we computed the maximum value of the curve of PSTH in the late phase (PSTH_late peak_). To quantify the reproducibility of the evoked response, we computed the reliability, defined as the fraction of total spikes that occurred during “events” (periods in which the firing rate was above 3 times the value of MFR during a period of no-stimulation), according to Jimbo *et al.* (1999) and Mainen and Seinowski (1995).

#### 3. Statistical analysis

Statistical analyses were performed using MATLAB (The MathWorks, Natick, MA, USA). We evaluated the data distribution exploiting the Kolmogorov–Smirnov normality test. Since the data were not normally distributed, we performed the non-parametric Kruskal–Wallis test. To determine statistically significant differences, p values < 0.05 were considered significant. In the text, the values are reported as mean ± standard deviation, unless differently stated.

## SUPPLEMENTARY MATERIAL

See the supplementary material for a description of the parameters computed from the acquired electrophysiological signals, further qualitative and quantitative analysis on the PSTH, and the tables of the performed statistical analysis.

## Data Availability

The data that support the findings of this study are available from the corresponding author upon reasonable request.
